# Diseases of the Heart and Circulation

**Published:** 1904-01-16

**Authors:** 


### DISEASES OF THE HEART AND CIRCULATION.

(*Concluded from page* 261.)

Ogle[4] has recently analysed the records of cases of malignant endocarditis published in the *Lancet* and *British Medical Journal* to find out whether the use of antistreptococcic serum has been followed by good results or not. He comes to the following conclusions : That the gravest symptoms, combined with streptococcic infection, even of the blood stream, are not incompatible with recovery if treated by injections of antistreptococcic serum. This is true also in malignant endocarditis, though the chances are probably less favourable on account of the colony of micrococci involved in the vegetations in constant contact with the blood stream. In malignant endocarditis staphylococci are frequent, or a mixed infection of staphylococci and streptococci. If an examination of the blood be negative it is prudent to use injections of both antistreptococcic and antistaphylococcic serum. In the use of these sera it has to be remembered that they are germicidal and not antitoxic in their action, thus differing, for instance, from antidiphtheritic serum which only neutralises toxic products.

**Abdominal Aneurysm.**—Dickinson[5] points out that aneurysms of the abdominal aorta or of the commencement of the cœliac axis or superior mesenteric artery are far more prone to rupture than thoracic aneurysms, in fact this is the almost invariable end. The extravasation generally takes place into the loose cellular tissue behind the peritoneum in the iliac and lumbar region, on one or both sides. The blood may coagulate and form for a time a diffuse false aneurysm. The symptoms are those of hæmorrhage and agonising pain due to stretching of the peritoneum. If the patient survives the diffuse aneurysm forms a large deep-seated fluctuating tumour, generally on the left side, which but for its sudden appearance and the presence of pulsation might be taken for an abscess. These diffuse aneurysms always progressively enlarge and commonly burst into the peritoneal cavity. External rupture does not occur. Nearly half the number of abdominal aneurysms rupture directly or indirectly into the peritoneal cavity, causing sudden death. Rupture into the alimentary canal is rare ; if it takes place it is usually into the duodenum, and is rapidly fatal with profuse hæmatemesis. Fifteen per cent. of cases rupture upwards into one of the pleural cavities, mainly the left. The blood passes by way of the retroperitoneal tissue through a weak space in the diaphragm—in front of the transversalis fascia and ligamentum arcuatum externum. Compression of the corresponding lung and displacement of the heart take place, and the event is generally fatal within a few hours, though in some cases not for some days. Thus a patient was admitted for dorsal pain and spastic gait—this was supposed to be due to spinal caries. Then a fluctuating non-pulsatile tumour developed in the left loin together with signs of fluid in the pleura above. This looked like a descending empyema in connection with spinal disease. Post-mortem examination showed a ruptured abdominal aneurysm and large effusion of blood into the pleura.

**Pulsatile Aorta.**—Huchard[6] states that the condition of pulsatile aorta is frequently overlooked or is erroneously diagnosed as aneurysm. In fact the aorta does show a slight amount of dilatation, and in some cases a slight blowing murmur is heard over it, but the chief point of distinction from aneurysm is that the aorta projects in front only, there being no lateral expansion. The pain occurs in the form of crises which may last for some weeks and disappear for long periods. These crises occur in neuropathic subjects who suffer from gastro-intestinal disorder, and also in exophthalmic goitre. The cause of the disorder is obscure, for neither aneurysm or aortitis is present. The treatment is chiefly sedative, and slight vaso-constrictors may be employed. Huchard recommends a pill containing extract of convallaria, hydrobromide of quinine, and ergotin. It may perhaps be recalled that Wade found good results from the use of vaso-dilators, for instance, $\frac{1}{8}$ to $\frac{1}{4}$ grain of nitroglycerine three times a day.

[4] Lancet, March 14. [5] Clin. Jour., March 4. [6] Brit. Med. Jour., Feb. 2.

